# The Treatment of Headaches

**Published:** 1893-03-18

**Authors:** 


### THE BATH EYE INFIRMARY.

### The Treatment of Headaches.

It is only within recent years that the treatment of headaches by ophthalmic surgeons has been recognised as a scientific procedure in certain cases. Formerly the idea would have been scouted that a pair of spectacles, could do anything beyond improving vision. The practitioner of to-day, however, knows that there are other benefits to be derived from their use, and before resorting to drugs for the cure of chronic headaches, carefully finds out the refraction of his patient's eyes. Surprise is often expressed by patients when they are told of the possibility of their headaches being due to malformation of their eyes. They believe their sight to be perfect, both for distant objects and for reading; in fact, many of them pride themselves on being very long sighted, little knowing that their long sight is consistent with a high degree of hypermetropia. Others, again, well advanced in years, boast that they can read the smallest type at the usual distance without the aid of glasses—a sure and certain sign of myopia.

It may be laid down as an axiom that the patient who has never had his vision estimated is in absolute ignorance of its condition. And herein lies the difficulty in dealing with these patients, for as they have nothing to complain of in their eyes they insist on having something given to them in a six-ounce phial to cure their headache, and that this latter can be in any degree connected with eye-strain is quite beyond their comprehension. In many cases the fact that the headache is ocular in origin cannot be arrived at by the nature of the symptoms or by their locality. Sometimes the pain is said to be situated in the eyes themselves, or at the back of them; at other times in various parts of the head, temporal, parietal, vertical, or occipital. Its character may be described as throbbing, dull and heavy, or neuralgic, thus resembling headaches due to a variety of causes. It may be constant or periodic, or irregularly recurrent. Prolonged or unusual use of the eyes may be the starting of a headache that is migrainous in type. The patient who suffers from headache after visiting a picture gallery or a theatre but not after going to a ball or to church, is almost invariably the subject of ocular cephalalgia; but in most cases the diagnosis cannot be so easily made. Conversely, it does not follow because a patient suffers from headache when there is no obvious strain on the eyes that therefore his trouble is not due to refractive error, for the hypermetrope cannot even use his eyes for any purpose without some amount of accommodative effort.

At the Bath Eye Infirmary it is customary to atropize all patients who come for treatment in consequence of headache, unless from age or other circumstances it seems inadvisable to do so. Mr. Beaumont is very emphatic on the necessity of doing so, as he considers that if good results are to follow it is absolutely essential to perfectly correct the errors of refraction. Ametropia that is only partially corrected will often only proportionately, or it may be not at all, relieve the headache, for it is a well-recognised fact that it is frequently the errors of low degree that are associated with the most severe headaches. It is generally considered that the condition of refraction that is most often associated with headache is hypermetropia or hypermetropic astigmatism, but myopia is not infrequently also a cause. The myope, however, usually does not suffer so severely unless he is constantly overusing his eyes for fine print or other close work, whereas the hypermetrope suffers even when he is not so doing, the fact being that he is unconsciously using his accommodation all day long for seeing objects which the emmetrope sees without any muscular effort. The fact that he can thus see apparently as well as the emmetrope leads him to believe that his sight is perfect. The condition may be roughly compared to that of two men carrying cans of milk, one of whom carries them suspended from a yoke over his shoulders, thus requiring but little muscular effort, whilst the other one carries them in his hands, when the flexor muscles soon tire. Each may accomplish his day's work, but the one will do so with much less fatigue than the other. So with the hypermetrope, all he requires is a pair of spectacles to take the place of the milkman's yoke, and then the ciliary muscle will be saved its unnecessary effort, and the reflex headache will not be excited.

Ocular headache that has existed for a long period is not usually cured at once by the adoption of correcting lenses, for when the tendency has been long established an extended period of treatment is required before a cure can be effected. Patients should not be allowed to throw aside their glasses after a week or two of use if they find the headaches continue, but they should be encouraged to persevere with the certain hope that if the real cause is an error of refraction so surely will they be eventually relieved. In the whole realm of ophthalmology there is no more certain fact than this, and the practitioner whose acumen has first suggested that the eyes should be tested will be rewarded both by the cure and by the gratitude of his patient. There is something very mysterious to the ignorant in the cure of a headache by a pair of spectacles, and that which is mysterious is often looked upon as almost miraculous, *ergo*, the surgeon is a worker of wonders. Formerly patients suffering from ocular headaches went from specialist to specialist, seeking vainly for relief, the neurologist getting the lion's share of them. Any slight deviation from the normal standard of health in any organ was dosed and drugged in vain, till the sufferer at length resigned himself to bear, with what fortitude he could, that which appeared to be incurable. It is po ssible that many a young student has had a career that might have led to professional eminence cut short by headaches, and has emigrated to the colonies as the only occupation that could be suggested for him as a means of livelihood when a pair of spectacles might have made him a Bishop, a Lord Chancellor, or a Prime Minister.

The following notes, taken from the records of the Bath Eye Infirmary, are types of constantly-recurring cases: 1. A nursery governess at thirty-four had been almost constantly employed in teaching and sewing for sixteen years, during which time she never went a week without suffering from what she described as a "sick" headache. The only relief she could get was obtained by going to bed for a couple of hours in a dark room. She was wearing —4D, which had been ordered for her by an optician, and with which she said she could see well at a distance. For near work she did not wear glasses, as she said her sight was so "strong" that she could do the finest work, and she was quite sure that her headaches were not connected with her eyes. Examination under atropine disclosed compound myopic astigmatism, —2D in the horizontal meridian, and —4D in the vertical. Her refraction was fully corrected, and she was told to use the glasses for near work as well as far. Six months later she returned, stating that she rarely had a headache.

2. A trained nurse at 26, who had been a victim to headaches from her earliest schooldays, complained that in consequence thereof it was almost impossible for her to do any night nursing. The pains were of a neuralgic character in the temples, and she had taken much medicine in her vain endeavour to get relief. The only drug which appeared to afford her any ease was croton chloral. She was certain there was nothing wrong with her sight, and with both eyes open she could read some letters of $\frac{6}{6}$. Under atropine her vision was $\frac{6}{12}$ + 1 by axis vertical $\frac{6}{6}$. After a considerable amount of persuasion she consented to wear full corrective glasses, when her headaches soon ceased to trouble her, and in twelve months she had quite lost them. It would be useless detailing more cases; but these patients follow each other in the out-patient department of every ophthalmic institution in a ceaseless stream, giving monotonously similar accounts of their symptoms, and often ending with a peroration which is highly eulogistic of their own powers of vision, both physical and mental, and which is full of contemptuous pity for the fads of the medical man, to oblige whom they have condescended to allow the oculist to examine their sight.

